# [Retracted] 2’,4’-dihydroxychalcone, a flavonoid isolated from *Herba oxytropis*, suppresses PC-3 human prostate cancer cell growth by induction of apoptosis 

**DOI:** 10.3892/ol.2026.15809

**Published:** 2026-08-11

**Authors:** Yuqing Sheng, Mingchang Zou, Yan Wang, Qiheng Li

Oncol Lett 10: 3737–3741, 2015; DOI: 10.3892/ol.2015.3795

Following the publication of the above paper, it was drawn to the Editor's attention by a concerned reader that, regarding the western blots shown in Fig. 6 on p. 3740, a pair of the blots in Fig. 6A were overlapping with those featured in Fig. 6C; in addition, the Caspase-3 blots in Fig. 6A were strikingly similar to the PTEN blots featured in Fig. 6C, albeit with the lower bands of the doublets shown for Caspase-3 conspicuously absent. Thirdly, it was noted that the authors had presented PTEN western blotting results in PC-3 cells, which was an unexpected finding given that it has been demonstrated previously that the PC-3 cell line is known to have a homozygous deletion in the PTEN gene, and is therefore considered to be effectively PTEN-null. Furthermore, upon performing an independent examination of the data in this paper in the Editorial Office, it came to light that the p27Kip1 and PTEN bands in Fig. 1B closely resembled each other in terms of their overall shape, although the PTEN bands appeared darker, and it was conceivable that the same data may have been photographed after a longer exposure time.

In response to the queries raised by the concerned reader, the authors reported that they no longer had acccess to the all the original data pertaining to the western blots shown in Fig. 6. Furthermore, in light of the PC-3 cell line query, the authors proposed that the PTEN data be removed from the paper; however, in the original article, PTEN was presented as a central mechanistic biomarker, and the Editor considered that the removal of these data would have too substantive an effect on the interpretation of the study; in this situation, the authors were asked to provide further findings supporting the mechanism proposed in the study in the absence of PTEN-related results.

Although the authors did undertake further experimental work, after carefully considering the various problems associated with the PTEN-related aspect of the paper, and also after the identification of the various potential anomalies concerning the presentation of the western blots in Fig. 6, the Editor of *Oncology Letters* has decided that this paper should be retracted from the Journal on account of a lack of confidence in the presented data. The authors were asked for an explanation to account for these concerns, but the Editorial Office did not receive a reply. The Editor apologizes to the readership for any inconvenience caused.

